# FIBTEM Improves the Sensitivity of Hyperfibrinolysis Detection in Severe Trauma Patients: A Retrospective Study Using Thromboelastometry

**DOI:** 10.1038/s41598-020-63724-y

**Published:** 2020-04-24

**Authors:** Il-Jae Wang, Sung-Wook Park, Byung-Kwan Bae, Sung-Hwa Lee, Hyuk Jin Choi, Sung Jin Park, Tae Young Ahn, Tae Sik Goh, Min Jee Lee, Seok Ran Yeom

**Affiliations:** 1 Department of Emergency Medicine, Pusan National University Hospital, Pusan National University, School of Medicine, Busan, Korea; 2 Biomedical Research Institute, Pusan National University Hospital, Busan, Korea; 3 Department of Neurosurgery, Pusan National University Hospital, Busan, Korea; 4 Department of Trauma Surgery, Pusan National University Hospital, Busan, Seoul Korea; 5 Department of Orthopedic Surgery, Pusan National University Hospital, Busan, Seoul Korea; 6 Department of Emergency Medicine, Pusan National University Yangsan Hospital, Gyeongsangnam-do Yangsan, Korea

**Keywords:** Outcomes research, Risk factors

## Abstract

Rotational thromboelastometry (ROTEM) can only detect high-degree hyperfibrinolysis (HF), despite being frequently used in trauma patients. We investigated whether considering FIBTEM HF (the presence of maximal lysis (ML) > 15%) could increase ROTEM-based HF detection’s sensitivity. This observational cohort study was performed at a level 1 trauma centre. Trauma patients with an Injury Severity Score (ISS) > 15 who underwent ROTEM in the emergency department between 2016 and 2017 were included. EXTEM HF was defined as ML > 15% in EXTEM. We compared mortality rates between EXTEM HF, FIBTEM HF, and non-HF patient groups. Overall, 402 patients were included, of whom 45% were men (mean age, 52.5 years; mean ISS, 27). The EXTEM HF (n = 37), FIBTEM HF (n = 132), and non-HF (n = 233) groups had mortality rates of 81.1%, 22.3%, and 10.3%, respectively. The twofold difference in mortality rates between the FIBTEM HF and non-HF groups remained statistically significant after Bonferroni correction (P = 0.01). On multivariable Cox regression analysis, FIBTEM HF was independently associated with in-hospital mortality (adjusted hazard ratio 2.15, 95% confidence interval 1.21–3.84, P = 0.009). Here, trauma patients with FIBTEM HF had significantly higher mortality rates than those without HF. FIBTEM be a valuable diagnostic method to improve HF detection’s sensitivity in trauma patients.

## Introduction

Trauma is a major cause of death in people under the age of 40 years, and bleeding is the leading cause of preventable death in trauma patients^1,2^. More than 30% of haemorrhagic trauma patients present with coagulopathy at the emergency department (ED). In comparison with patients without coagulopathy, those with coagulopathy have increased risks of multiple organ failure, massive transfusion, and death^3–5^. Trauma-induced coagulopathy is a complex condition that is affected by various factors, such as haemodilution, hypothermia, fibrinolysis, activated protein C, endothelial changes, and platelet dysfunction^5–8^.

Hyperfibrinolysis (HF) isa central feature of traumatic coagulopathy and is defined as an abnormal increase in the degree of fibrinolytic activity compared to physiological fibrinolysis^9,10^. Since HF is associated with high mortality rates and massive bleeding, its early detection is important in the treatment of trauma patients. However, it is difficult to diagnose HF in a clinically relevant time frame^11,12^. Currently, viscoelastic haemostatic assays (VHAs), such asthromboelastography (TEG) and rotational thromboelastometry (ROTEM),are the only tools used for the detection of HF within a clinically relevant time frame^11,13^. However, VHA only detects massive fibrinolytic activation and is insensitive in the identification of non-massive HF^14^.

In the FIBTEM test, the contribution of plateletsto clot formation is inhibited by cytochalasin-D^13^. Because platelets retain large amounts of plasminogen activator inhibitor and because platelet-rich thrombus is resistant to tissue plasminogen activator (tPA)-mediated fibrinolysis^15,16^, we hypothesized that the sensitivity of HF detection is improved with the use of the FIBTEM test. This study aimed(a) to compare mortality rates between FIBTEM HF andnon-HF patient groups and (b) to determine whether FIBTEM HF is independently associated with mortality.

## Results

### Baseline characteristics

During the study period, a total of 426 severe trauma patients (ISS > 15) underwent the ROTEM test. The exclusion criteria were age <15 years (n = 7) and presentation at our hospital 12 hours after trauma occurrence (n = 17). The final study population consisted of 402 patients. The participants’ mean age was 52.5 years, 45% of them were male, and their median ISS was 27. The most commonly observed injury mechanism was traffic accident (58%), followed by falling to a lower level (27%). Seventy-nine patients (19.7%) underwent MT, and the overall in-hospital mortality rate was 20.6%. The baseline characteristics of the patients are summarized in Table 1.

**Table 1:** Characteristics, vital signs, and outcomes with EXTEM HF group, FIBTEM HF group, and non-HF group.

Variable	Overall (n = 402)	Non-HF (n = 233)	FIBTEM HF (n = 132)	EXTEM HF (n = 37)	P value
Male, n (%)	183 (45.5)	100 (42.9)	61 (46.2)	22 (59.5)	0.169
Age (y), mean (SD)	52.50 (17.95)	53.26 (17.70)	51.70 (18.23)	50.59 (18.79)	0.592
**Injury mechanism, n (%)**					
Traffic accident	234 (58.2)	129 (55.4)	84 (63.6)	21 (56.8)	0.307
Falling to a lower level	110 (27.4)	63 (27.0)	36 (27.3)	11 (29.7)	
Penetrating	8 (2.0)	7 (3.0)	0 (0.0)	1 (2.7)	
Others	50 (12.4)	34 (14.6)	12 (9.1)	4 (10.8)	
SBP, mean (SD)	99.95 (43.01)	107.35 (35.68)	101.14 (43.15)	49.11 (51.07)^a,b^	<0.001
Heart rate, mean (SD)	94.96 (29.36)	97.33 (25.41)	93.95 (27.82)	83.65 (49.57)	0.170
RTS, median [IQR]	11.00 [9.00, 12.00]	11.00 [10.00, 12.00]	11.00 [9.00, 12.00]^a^	7.00 [1.00, 8.00]^a,b^	<0.001
ISS, median [IQR]	27.00 [22.00, 34.00]	25.00 [21.00, 30.00]	27.00 [22.00, 34.25]^a^	34.00 [26.00, 41.00]^a,b^	<0.001
4 h RBC, median [IQR]	1.00 [0.00, 4.75]	1.00 [0.00, 3.00]	2.00 [0.00, 6.00]^a^	10.00 [5.00, 19.00]^a,b^	<0.001
MT, n (%)	79 (19.7)	30 (12.9)	27 (20.5)	22 (59.5)^a,b^	<0.001
In hospital mortality, n (%)	83 (20.6)	24 (10.3)	29 (22.0)^a^	30 (81.1)^a,b^	<0.001

HF, hyperfibrinolysis; SD, standard deviation; SBP, systolic blood pressure; RTS, revised trauma score; IQR, interquartile range; ISS, injury severity score; 4 h RBC, packed red blood cell units for 4 hours; MT, massive transfusion.

^a^P < 0.05 compared to non-HF group, ^b^P < 0.05 compared to FIBTEM HF group.

### Comparison of the EXTEM HF, FIBTEM HF, and non-HF groups

The highest proportion of patients belonged to the non-HF group (58.0%), followed by the FIBTEM HF group (32.8%) and the EXTEM HF group (9.2%). All patients in the EXTEM HF group showed FIBTEM ML > 15%. There were no statistical differences in terms of age, sex, or injury mechanism between the groups.

**Figure 1:**
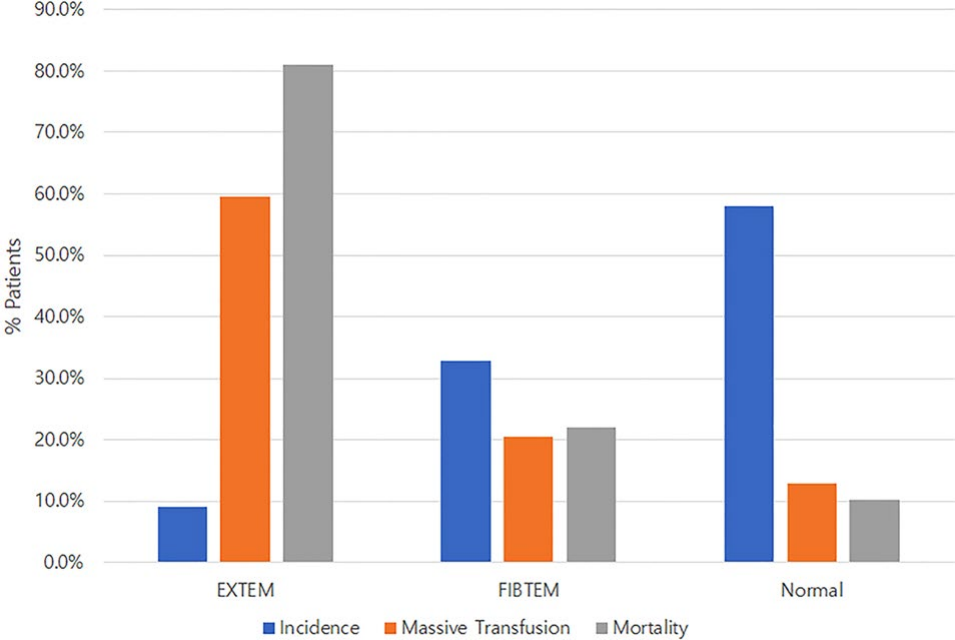
Incidence, massive transfusion, and in-hospital mortality in the EXTEM HF, FIBTEM HF, and non-HF groups. HF: hyperfibrinolysis.

Compared to the non-HF group, the FIBTEM HF and EXTEM HF groups hadhigher median ISSs (25 vs. 27 vs. 34, P < 0.001) and received MT more frequently (12.9% vs. 20.5% vs. 59.5%, P < 0.001). The mortality rate was also significantly different between the groups (P < 0.001). The mortality rate in the EXTEM HF group was the highestand was about eighttimes higher than that in the non-HF group (81.1% vs. 10.3%). The mortality in the FIBTEM HF group was about twotimes higher than that in the non-HF group (Fig. 1); this difference was statistically significant after Bonferronicorrection (22.3 vs. 10.3%, P = 0.01) (Table 1).

Significant differenceswere observed in laboratory values and ROTEM parametersacross the groups (Table 2). The EXTEM HF group showed the lowest platelet and fibrinogen values, followed by the FIBTEM HF groupand non-HF group. After performing Bonferroni corrections, the FIBTEM HF group continued to have significantly lower platelet (P = 0.019) and fibrinogen (P < 0.001) values than the non-HF group. The PT INR and aPTTvalues were the highest in the EXTEM HF group, while the FIBTEM HF group showed higher PT INR and aPTTvalues than the non-HF group.

**Table 2:** Laboratory value and ROTEM parameter with EXTEM HF group, FIBTEM HF group, and non-HF group.

Variable	Overall (n = 402)	Non-HF (n = 233)	FIBTEM HF (n = 132)	EXTEM HF (n = 37)	P value
Fibrinogen, median[IQR]	193.80 [140.67, 234.60]	214.00 [179.40, 265.85]	159.20 [122.17, 203.62]^a^	125.70 [76.30, 172.95]^a,b^	<0.001
PTINR, (median[IQR]	1.08 [0.99, 1.23]	1.03 [0.97, 1.15]	1.11 [1.02, 1.32]^a^	1.43 [1.25, 1.84]^a,b^	<0.001
aPTT, median [IQR]	30.20 [26.40, 36.75]	29.10 [25.60, 33.10]	30.80 [26.75, 39.60]^a^	55.40 [39.80, 116.40]^a,b^	<0.001
Hb, median[IQR]	12.50 [10.90, 14.20]	12.60 [11.00, 14.30]	12.40 [10.88, 14.12]	12.10 [9.40, 13.80]	0.141
Platelet, median [IQR]	212.50 [168.25, 258.25]	225.00 [178.00, 262.00]	199.00 [148.75, 251.25]^a^	172.00 [103.00, 193.00]^a,b^	<0.001
pH, median[IQR]	7.38 [7.29, 7.42]	7.39 [7.33, 7.43]	7.36 [7.29, 7.41]^a^	7.22 [7.01, 7.32]^a,b^	<0.001
Base Excess, median[IQR]	−2.80 [−7.30, −0.20]	−2.05 [−6.23, 0.80]	−3.50 [−7.55, −0.85]^a^	−11.80 [−16.12, −6.75]^a,b^	<0.001
HCO3, median [IQR]	22.30 [18.80, 24.80]	22.70 [19.85, 25.30]	21.90 [18.55, 24.40]	17.80 [12.57, 20.02]^a,b^	<0.001
EXTEM_CT, median [IQR]	59.00 [52.00, 73.00]	55.00 [50.00, 64.00]	64.00 [56.00, 78.00]^a^	88.00 [71.00, 135.00]^a,b^	<0.001
EXTEM_α, median [IQR]	71.00 [65.00, 75.00]	72.00 [69.00, 76.00]	69.00 [62.00, 73.00]^a^	60.00 [51.00, 67.00]^a,b^	<0.001
EXTEM_MCF,median [IQR]	60.00 [54.00, 63.00]	62.00 [58.00, 65.00]	57.00 [51.00, 61.00]^a^	40.00 [28.00, 50.00]^a,b^	<0.001
FIBTEM_CT, median [IQR]	63.00 [51.00, 92.00]	57.00 [49.00, 71.75]	77.00 [55.00, 129.25]^a^	102.00 [77.00, 510.00]^a,b^	<0.001
FIBTEM_α, median [IQR]	49.00 [0.00, 67.00]	60.00 [42.00, 69.00]	0.00 [0.00, 54.50]^a^	0.00 [0.00, 16.00]^a^	<0.001
FIBTEM_MCF (median [IQR])	11.00 [7.00, 15.00]	13.00 [10.00, 17.00]	9.00 [6.00, 12.00]^a^	6.00 [4.00, 9.00]^a,b^	<0.001
FIBTEM_ML (median [IQR])	6.00 [3.00, 9.75]	100.00 [100.00, 100.00]	28.00 [20.00, 45.00] ^a^	100.00 [100.00, 100.00] ^a,b^	<0.001

HF, hyperfibrinolysis; IQR, interquartile range; PT_INR, prothrombin time and international normalized ratio; aPTT, activated partial thromboplastin time; Hb, hemoglobin; pH, potential hydrogen; HCO3, bicarbonate.

^a^P < 0.05 compared to non-HF group, ^b^P < 0.05 compared to FIBTEM HF group.

### Survival analysis

**Figure 2:**
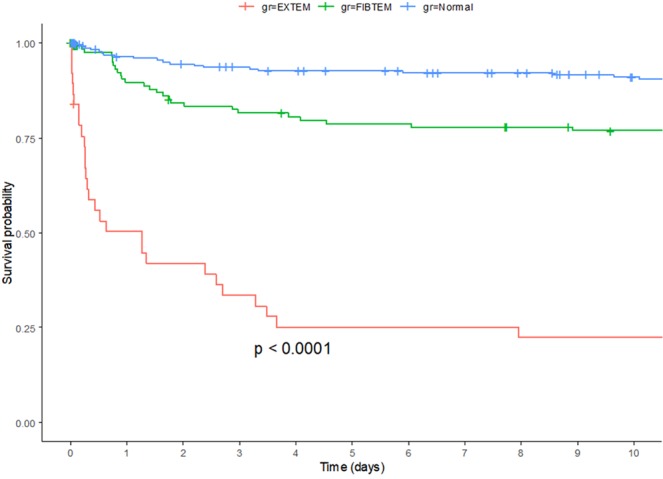
Kaplan–Meier survival analysis in the EXTEM HF, FIBTEM HF, and non-HF groups. HF: hyperfibrinolysis.

Kaplan–Meier curves and a log rank test showed that that mortality increased from the non-HF to the FIBTEM HF and EXTEM HF groups (Fig. 2). The results of the univariableand multivariable Cox regression modelsare shown in Table 3. In the multivariable Cox regression model, FIBTEM HF was independently associated with in-hospital mortality after adjustment for age, sex, injury mechanism, fibrinogen, ISS, and platelet count. The adjusted hazard ratio (aHR) indicated significantly elevated in-hospital mortality in the FIBTEM HF group, compared to thatin the non-HF group (aHR 2.153, 95% confidence interval 1.208–3.837, P = 0.009).

**Table 3:** Cox regression for in-hospital mortality.

Variables	Unadjusted HR [95% CI]	Unadjusted p value	Adjusted HR [95% CI]	Adjustedp value
**HF**				
non-HF	Reference		Reference	
EXTEM	15.775 [9.163, 27.157]	<0.001	8.158 [4.161, 15.997]	<0.001
FIBTEM	2.423 [1.410, 4.164]	0.001	2.153 [1.208, 3.837]	0.009
Male gender	1.426 [0.927, 2.193]	0.107	0.995 [0.627, 1.578]	0.982
Age	1.017 [1.004, 1.030]	0.010	1.023 [1.009, 1.038]	0.001
**Injury mechanism**				
penetrating	Reference		Reference	
Traffic accident	1.080 [0.666, 1.752]	0.754	1.080 [0.666, 1.752]	0.921
Falling to a lower level	0.894 [0.439, 1.823]	0.759	1.318 [0.167, 10.405]	0.793
Others	0.541 [0.075, 3.923]	0.543	1.039 [0.121, 8.905]	0.972
SBP	0.982 [0.977, 0.987]	<0.001	0.993 [0.987, 0.999]	0.016
ISS	1.054 [1.036, 1.072]	<0.001	1.040 [1.018, 1.062]	0.000
Fibrinogen	0.994 [0.991, 0.997]	<0.001	1.000 [0.998, 1.003]	0.717
PT_INR	1.337 [1.235, 1.448]	<0.001	1.164 [1.051, 1.289]	0.003

HR, hazard ratio; CI, confidence interval; HF, hyperfibrinolysis; SBP, systolic blood pressure;ISS, injury severity score; PT_INR, prothrombin time and international normalized ratio.

## Discussion

In this study, we categorized trauma patients into the EXTEM HF, FIBTEM HF, and non-HF groups. Although the incidence of EXTEM HF was low (9%), the mortality rate of patients with EXTEM HF was extremely high (81%). Patients with FIBTEM HF accounted for one-third of the study population, and their mortality was more than twice as that of patients in the non-HF group (22% vs. 10%). The FIBTEM HF group underwent MT more commonly and had a worse level of coagulopathy than the non-HF group. In the multivariable Cox regression model, FIBTEM HF was identified as an independent risk factor for in-hospital mortality.

A number of severe trauma patients have disproportionately increased fibrinolytic activity, and HF is an important component of acute traumatic coagulopathy^11,12^. VHAs, such as TEG and ROTEM, provide general information fromclot formation to clot lysis and are the only tools for the diagnosis of HF within an appropriate time frame^17^. However, previous studies have shown that VHAs have low sensitivity for HF detection^4,14^. In 2013, Raza *et al*.^14^ classified patients who presented to a major trauma centre into three groups, based on their fibrinolytic activity: a normal group (plasmin-α2 antiplasmin [PAP] < 1,500 µg/L), a moderate group (PAP > 1,500 µg/L and EXTEM ML < 15%), and a severe group (PAP > 1,500 µg/L and EXTEM ML > 15%). More than half of the patients (57%) showed moderate fibrinolytic activity, and their 28-day mortality rate was more than 12times higher than that observed in the normal fibrinolytic activity group. Only 5% of cases with severe fibrinolytic activity could be detected using ROTEM analysis, suggesting that a large number of cases with fibrinolytic activity were undetected by ROTEM,resulting in a noticeably high mortality.

Platelets play a key role in haemostasis, and previous studies have demonstrated that thrombocytopenia and platelet hypofunction are closely related to mortality^12,18–21^. Plateletsare also related to fibrinolysis, and Franz *et al*. showed that platelet-rich plasma is more resistant to tPA-induced fibrinolysis than platelet-poor plasma^22^. Based on these observations, we focused on FIBTEM,in which platelet function is blocked. In our study, similar to previous studies, the prevalence of EXTEM HF was low, but the associated mortality was extremely high^14,23^. The number of patients with FIBTEM HFwas more than three times higher than the number of patients with EXTEM HF, and the mortality rate was more than twice as high as in the non-HF group. Among the patients with FIBTEM HF, MT was performed more frequently, and such patients had a worse degree of coagulopathy.

Several studies have usedthe FIBTEM test in trauma patients^3,24,25^. Schöchl *et al*. reported that FIBTEM MCF and FIBTEM A10 had a high predictive value for MT in trauma patients^3^. Hagemo *et al*. showed that a FIBTEM CA5 threshold of ≤9 mm led to the detection of the need for MT in 77.5% of patients^24^. Although not intended for injury patients, Harr *et al*. found that FIBTEM detects fibrinolysis faster than INTEM and EXTEM^26^. However, to the best our knowledge, this is the first study to use FIBTEM HF to improve the sensitivity of HF detection.

Tranexamic acid (TXA) is a synthetic lysine analoguethat prevents the conversion of plasminogen to plasmin. It is used in the only evidence-based method available to treat HF^11^. However, there is a controversy regarding indications for TXA administration. Recent studies have focused on hypofibrinolysis (fibrinolytic shutdown, SD), demonstrating that SD is the most common fibrinolytic state in severe trauma patients and that the mortality rate in such patients is higher than that in patients in a physiologic fibrinolytic state^27,28^. There is concern surrounding the increases in mortality rate and frequency of thrombotic complications when TXA is administered to patients with SD who are already in a hypofibrinolytic state^18,29^. Furthermore, unlike the CRASH trial, in which the use of TXA was emphasized, opposing results were observed in clinical research studies,in which the use of TXA enhanced the occurrence of thrombotic events^30–32^. We believe that FIBTEM HF may have potential for use in selective TXA administration; however, additional research on the topic is needed.

Our study had several limitations. First, owingto its single-centredesign, generalizationof the results is difficult. As we only included severe trauma patients (ISS > 15), the applicability of FIBTEM HF in patients with a less severe condition is uncertain. Further multicentreinvestigations are needed to demonstrate the clinical value of FIBTEM HF. Second, there are several laboratoryvalues that can be used to measure fibrinolysis, such as euglobulin clot lysis time, plasmin-antiplasmin complex level, and plasminogen activator inhibitor-1 level. Although these tests are time-consuming and of limited availability in most trauma centres, the extent of fibrinolysis can be assessed better when analysed in combination with VHA.

In conclusion, although previous studies have emphasized the utility of VHA in the detection of HF in trauma patients, VHA can only detect high fibrinolytic activity. In the current study, we focused on FIBTEM HF and observed that mortality rates and coagulopathy were worse among patients with FIBTEM HF than among patients without HF. We suggest that FIBTEM may be used as a method of improving the sensitivity of HF detection in severe trauma patients.

## Materials and Methods

Institutional Review Board of our hospital approved this study and also waived the need for informed consent. All methods were performed in accordance with the relevant guidelines and regulations.

### Study design and setting

This retrospective, observational, single-centre study included patients who presented to the trauma centre of a 1400-bed university-affiliated hospital in Pusan, Korea. Our trauma centre serves as a level 1 trauma centre for patients in Busan City and Gyeongsangnam-do Province. Almost 1,000 severe trauma patients (Injury Severity Score [ISS] > 15) present to our trauma centre annually.

### Participants

From January 2016 to December 2017, all injured patients who presented to the trauma centre’s ED with an ISS > 15 and who underwent the ROTEM test were included. The exclusion criteria were (a) age <15 years, (b) presentation at the hospital ≥12 hours after trauma occurrence, and (c) presence of a burn injury.

### Data collection and variables

We extracted data from the Korea Trauma Database (KTDB) and electronic medical records. The KTDB was created by the Korean Ministry of Health and Welfare in 2013 for the purpose of collecting information on trauma patients nationwide^33^. The collected data included data of age, sex, vital signs at trauma centre presentation, injury mechanism, revised trauma score at initial trauma centre presentation, transfusion of packed red blood cell within the first 4 and 24 hours of ED admission, ISS, massive transfusion (MT), and in-hospital mortality. Laboratory (fibrinogen, prothrombin time international normalized ratio [PT INR], activated partial thromboplastin time [aPTT], haemoglobin, platelet, pH, base excess, HCO_3_) and ROTEM data were also collected. Blood for ROTEM analysis and laboratory test was drawn within 15 minutes of initial ED presentation. Our primary outcome was in-hospital mortality, while the secondary outcome was MT. We defined MT as the transfusion of ≥10 U of packed red blood cells within 24 hours^34^.

### Viscoelastic testing

Viscoelastic haemostatic analysis was performed using ROTEM delta (TEM international GmbH, Munich, Germany). We used three ROTEM tests in the current study: EXTEM, FIBTEM, and APTEM. In the EXTEM test, clot formation is activated by a tissue factor. In the FIBTEM test, a platelet inhibitor (cytochalasin-D) is added, and the platelet contribution to clot formation is removed. The APTEM test includes a plasmin inhibitor, which is an antifibrinolytic agent and inhibits fibrinolysis. For each ROTEM test, the following data were collected: clotting time, clot formation time, maximum clot firmness (MCF), alpha angle, and maximum lysis (ML). We divided the patients into three groups: the non-HF group, EXTEM HF group, and FIBTEM HF group. We defined HF as the presence of EXTEM ML ≥ 15% and normal APTEM ML^11,33,35^. FIBTEM HF was defined as FIBTEM ML ≥ 15% and normal APTEM ML.

### Statistical analysis

For the continuous variables, normality was determined by a histogram and skewness >2. Normally distributed continuous variables were summarized in terms of means and standard deviations. Non-normally distributed continuous variables were summarized in terms of medians and interquartile ranges. We compared continuous variables using one-way analysis of variance or a Kruskal-Wallis test with a Bonferroni correction. Categorical variables were summarized in terms of counts and percentages and were compared using the Wilcoxon rank-sum test with Bonferroni correction. In-hospital survival was evaluated using Kaplan–Meier analysis, and a log rank test was performed to compare differences. Cox proportional hazards models were used to assess the independent effect of FIBTEM HF on in-hospital mortality. Baseline characteristics (sex, age, and injury mechanism) and previously suggested risk factors (PT INR, fibrinogen, and ISS) were included as covariates in the multivariable Cox regression model^6,36–38^. All statistical analyses were performed using R version 3.5.0 (R Foundation for Statistical Computing, Vienna, Austria). P values were two-sided, and a p value lower than 0.05 was considered statistically significant.

### Ethics approval and consent to participate

This study was approved by the Institutional Review Board of Pusan National University Hospital (1805-019-067). The need for written informed consent was waived as the data were analysed retrospectively and anonymously.

## Data Availability

The datasets used and/or analysed during the current study are available from the corresponding author on reasonable request.
